# Visceral leishmaniasis in the hills of western Nepal: A transmission assessment

**DOI:** 10.1371/journal.pone.0289578

**Published:** 2024-04-17

**Authors:** Surendra Uranw, Narayan Raj Bhattarai, Kristien Cloots, Lalita Roy, Keshav Rai, Usha Kiran, Uttam Raj Pyakurel, Bibek Kumar Lal, Sakib Burza, Suman Rijal, Prahlad Karki, Basudha Khanal, Epco Hasker

**Affiliations:** 1 Department of Internal Medicine, B.P. Koirala Institute of Health Sciences, Dharan, Nepal; 2 Department of Microbiology, B.P. Koirala Institute of Health Sciences, Dharan, Nepal; 3 Department of Public Health, Institute of Tropical Medicine, Antwerp, Belgium; 4 Tropical & Infectious Diseases Center, B.P. Koirala Institute of Health Sciences, Dharan, Nepal; 5 World Health Organization, Country Office for Nepal, Kathmandu, Nepal; 6 Epidemiology and Disease Control Division, Department of Health Services, Government of Nepal, Kathmandu, Nepal; 7 Faculty of Infectious Tropical Diseases, London School of Hygiene and Tropical Medicine, London, United Kingdom; 8 Drugs for Neglected Diseases Initiative, India Office, New Delhi, India; Virginia Commonwealth University, UNITED STATES

## Abstract

In Nepal, visceral leishmaniasis (VL) has been targeted for elimination as a public health problem by 2026. Recently, increasing numbers of VL cases have been reported from districts of doubtful endemicity including hills and mountains, threatening the ongoing VL elimination program in Nepal. We conducted a multi-disciplinary, descriptive cross-sectional survey to assess the local transmission of *Leishmania donovani* in seven such districts situated at altitudes of up to 1,764 meters in western Nepal from March to December 2019. House-to-house surveys were performed for socio-demographic data and data on past and current VL cases. Venous blood was collected from all consenting individuals aged ≥2 years and tested with the rK39 RDT. Blood samples were also tested with direct agglutination test, and a titer of ≥1:1600 was taken as a marker of infection. A *Leishmania donovani* species-specific PCR (SSU-rDNA) was performed for parasite species confirmation. We also captured sand flies using CDC light traps and mouth aspirators. The house-to-house surveys documented 28 past and six new VL cases of which 82% (28/34) were without travel exposure. Overall, 4.1% (54/1320) of healthy participants tested positive for *L*. *donovani* on at least one serological or molecular test. Among asymptomatic individuals, 17% (9/54) were household contacts of past VL cases, compared to 0.5% (6/1266) among non-infected individuals. *Phlebotomus argentipes*, the vector of *L*. *donovani*, was found in all districts except in Bajura. *L*. *donovani* was confirmed in two asymptomatic individuals and one pool of sand flies of *Phlebotomus (Adlerius)* sp. We found epidemiological and entomological evidence for local transmission of *L*. *donovani* in areas previously considered as non-endemic for VL. The national VL elimination program should revise the endemicity status of these districts and extend surveillance and control activities to curb further transmission of the disease.

## Introduction

Visceral leishmaniasis (VL), also called kala-azar (KA), is on the verge of elimination as a public health problem in the south-east Asia region, with a deadline currently set for 2026 [1]. In 2013, Nepal was the first country in the region to reach the elimination target of an annual incidence of less than one case per 10,000 population in all its officially endemic districts (i.e., districts where local *Leishmania* transmission has been documented, warranting the implementation of the national VL control program). In 2017, one of the non-endemic districts in the mountainous region breached the elimination target threshold and several other non-endemic districts have done so since then. Most of these districts with the presence of VL cases, later classified as endemic doubtful districts, are neither well prepared for patient management nor disease control efforts and surveillance [2]. Over the last decade, the VL case burden in Nepal has spread from east to west, from lowlands to hills and mountainous regions. The proportion of VL cases being reported from endemic doubtful districts has been steadily increasing, reaching >40% in 2020 (S1 Fig). If this trend represents a true increase, this will pose an important threat to the ongoing VL elimination target, warranting a re-assessment of the entire endemicity map and disease control approach in Nepal.

For a long time, it was assumed that all VL cases reported from these VL endemic doubtful districts were ‘imported cases’ (i.e., individuals that had been infected in other endemic doubtful districts and carried the disease to their districts of origin). This hypothesis was supported by the fact that VL has historically been a disease of the southern plain lowlands of Nepal called the *‘Terai’*, with a mild sub-tropical climate. Many other endemic doubtful districts in Nepal are located in hilly and even mountainous areas at high altitudes where the climate is generally considered unfavorable for the survival of the vector of VL, *Phlebotomus argentipes*. This assumption, however, is contradicted by the fact that many VL patients reported from these endemic doubtful districts have no apparent travel history to endemic areas including children who have never left their home village [3]. In addition, an outbreak investigation carried out by the B.P. Koirala Institute of Health Sciences (BPKIHS) in 2014 documented local transmission of *Leishmania* in six hilly districts in the eastern part of Nepal. This already illustrated that historically accepted assumptions about the ecological prerequisites for VL endemicity should be revisited [4].

**Fig 1:**
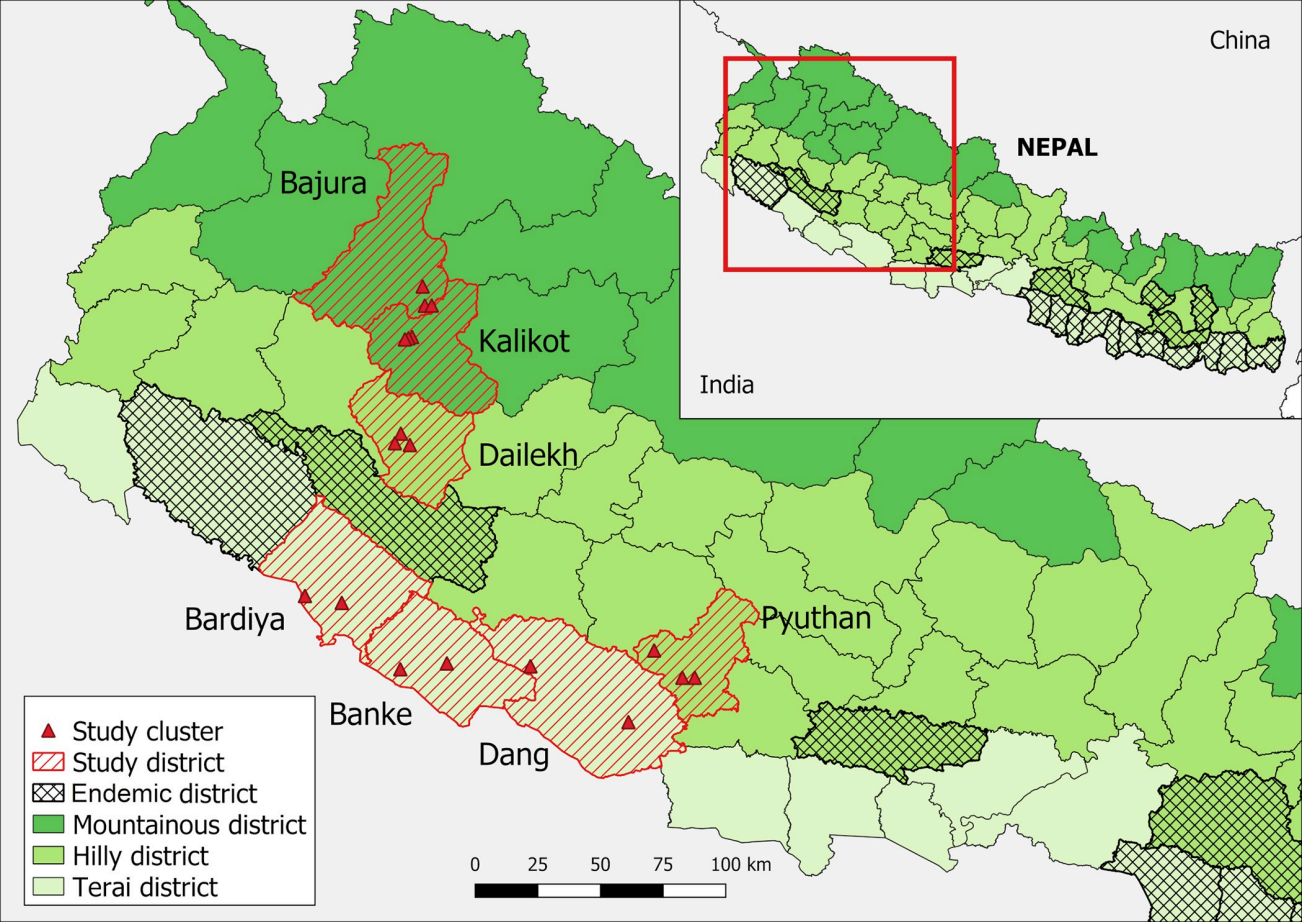
Location of the study districts. The map was produced with QGIS (version 3.10) with open access shapefile (New political and administrative boundaries Shapefile of Nepal—Datasets—Open Data Nepal).

A WHO pre-validation assessment conducted in 2017 pointed out that a better understanding of the full extent of VL endemicity is crucial for the validation of VL elimination in Nepal [5]. With this recommendation, we set out on a multidisciplinary survey to assess possibility of local transmission of *Leishmania* in seven selected endemic doubtful districts in western Nepal that were not covered by VL elimination program activities (Fig 1).

## Materials and methods

### Ethics statement

Ethical approval was granted to conduct this study from Institutional Review Committee of BPKIHS, Dharan, Nepal (Code No. IRC/1453/018). Community consent was obtained during meetings with local health facilities’ staff, village authorities, and village assemblies. Individual written informed consent was taken prior to blood sampling and interviewing. For children, a parent or guardian provided written informed consent. Permission to collect sand flies from households and cattle sheds was obtained from the heads of the households.

### Selection of study districts and clusters

We reviewed VL surveillance data of the Epidemiology and Disease Control Division (EDCD), Government of Nepal for the period of five years (January 2014 –December 2018). A total of seven districts namely Banke, Bardiya, Bajura, Dang, Dailekh, Kalikot and Pyuthan were selected for data collection based on the selection criteria: a) continuous reporting of VL cases during the last five years, b) increasing case load in recent years, c) increasing pediatric VL cases reported, and d) access to reach. Within each district, two to three clusters (part of a ward, smallest administrative unit) were selected based on the criteria: a) number of VL cases reported during each of the previous two years, and b) accessibility. The clusters selected for field work were: Mahendrapur-2 and Puraina-21 in Banke; Baida-10 and Santipur-5 in Bardiya; Budhinand-1, Jagganath-2 and Jagganath-5 in Bajura; Dhanauri-3, Lamahi-5 and Tulashipur-5 in Dang; Bhairabi-5, Dullu-11 and Guranse-4 in Dailekh; Baitee-8, Fukot-5 and Raskot-8 in Kalikot; and Airavati-4, Airavati-6 and Swargadwari-9 in Pyuthan district. The location of these study clusters is illustrated in Fig 1.

### Field work

The study had a descriptive cross- sectional design. The field work was carried out from March till December 2019. The approach used to assess local transmission was based on methodology used in a previous assessment and the national VL elimination program for deciding on VL endemicity since 2016 [2, 4]. The approach was based on three pillars: a) an epidemiological survey, aiming to trace past and present VL and post-kala-azar dermal leishmaniasis patients (PKDL—an infectious cutaneous sequel of VL), b) a serological and molecular survey aiming to identify asymptomatically infected individuals, and c) an entomological survey to assess presence of the (infected) vector.

### Epidemiological survey

We obtained community consent prior to the house-to-house survey in selected clusters. Each household was visited by a member of the field team and written informed consent was obtained from all individuals aged ≥18 years and assent in case of minors aged ≥2 to <18 years old. We screened all consenting individuals for VL, interviewed them if required for case validation and on travel history. We collected GPS coordinates of the households as well. Research assistants, trained on visually detecting macular, papular and nodular lesions with the help of a pictorial album, examined all enrolled past VL cases for PKDL like skin lesions. Suspected PKDL cases were referred to nodal dermatologists in a nearby tertiary care hospital for further case management (i.e., clinical examination, parasitological and histopathology test for PKDL confirmation). The investigators followed up the referred cases with the nodal dermatologists to obtain the final diagnosis.

*(i) Retrospective VL case validation*. We collected information on history of VL treatment, corroborated by prescriptions and case records from the health facility. In addition, we collected information on past VL deaths, which were defined as any deaths related to a febrile illness of ≥2 weeks in combination with at least one or more VL specific signs (*i*.*e*., enlargement (swelling) of the spleen and liver) as verified by a verbal autopsy.

*(ii) Assessment of travel history*. We asked all participants about their travel history or exposure to known VL endemic areas either in Nepal, India or Bangladesh within the two years prior to reporting first signs and symptoms of VL.

*(iii) Active case finding*. We tested individuals reporting fever for ≥2 weeks with the rK39 Rapid Diagnostic Test (RDT, In*B*ios International, Seattle, WA) and examined them for spleen enlargement. In case of a positive result with rK39 RDT, the individuals were referred to the nearest VL treatment center for further diagnosis and case management.

### Serological and molecular survey

We requested all individuals aged ≥2 years living in the study clusters to provide a blood sample. Trained laboratory technicians collected a 2 ml blood sample in an EDTA tube (for molecular analysis) and a serum tube (for serological analysis). In addition, an rK39 RDT test was done for each individual enrolled in the survey. The collected blood samples were transported in a cold box maintained at 4–8°C temperature until storage at -20°C at BPKIHS Dharan, Nepal for further analysis.

### Entomological survey

We collected sand flies during the expected peak season in Nepal (March–December) [6]. In each cluster, sand flies were captured indoors for two consecutive nights in twelve households. These households represented a combination of human dwellings, mixed dwellings, and cattle sheds, including houses of past or current VL cases. One CDC light trap was installed indoors in each dwelling and switched on between 6:00 pm and 6:00 am, after which the trapped sand flies were collected. The same process was repeated the following night. In addition, resting sand flies were collected by mouth aspirators from walls, poles or silos for 15–30 minutes in each dwelling in the early morning. Captured sand flies were separated from other insects and morphologically identified according to the regional keys [7, 8]. Female *Phlebotomus* spp. were pooled at household level in a cryotube with 80% alcohol for further molecular investigations to detect *Leishmania* sp.

### Laboratory analyses

#### Direct agglutination test

The blood samples were tested using a freeze-dried antigen of fixed, trypsin-treated and stained promastigotes of *L*. *donovani* 1-S obtained from the Institute of Tropical Medicine, Antwerp, Belgium as described by Jacquet *et al*. [9] and a serum dilution titer ≥ 1:1600 was taken as a marker of *L*. *donovani* infection [10].

#### PCR-based detection of *L*. *donovani complex*

DNA was extracted from individual human blood samples and pools of female sand flies of genus *Phlebotomus* (pooled per species at household level ranged from 1–20 in number) using the DNeasy Blood & Tissue Kit (Qiagen, Hilden, Germany). DNA from 200 μl blood or sand fly pool was eluted in 50 μl AE buffer. *L*. *donovani* species-specific PCR targeting on the small-subunit ribosomal DNA (SSU-rDNA) was performed on 2.5 μl DNA of each sample [11, 12]. The sense primer 18S-L-F5’-CGTAGTTGAACTGTGGGCTGTGC-3’, the anti-sense primer 18S-L-R 5’-ACTCCCGTGTTTCTT-GTTTCTTTGAA-3’ amplify the target and the probe FAM-CTGGTCGTCCCGTCCATGTCGGATT-BHQ1-ZEN detects the 115 bp sequence within the 18S rRNA gene of *Leishmania*. The 25 μl reaction mixtures contain 1X Go Taq Probe master mix (Promega, USA), 0.4 μM of each primer, 0.1 μM of probe (IDT, Singapore) and 0.1 mg/ml acetylated bovine serum albumin (Promega, USA). An initial denaturation step at 94°C for 15 min followed by 45 cycles of 94°C for 30 sec, 60°C for 30 sec and 72°C for 30 sec. The real time PCR amplification was conducted in 0.2 ml PCR tubes in Rotor Gene Q (Qiagen, Hilden, Germany). Pools of sand flies that were positive for the SSU-rDNA PCR were molecular typed by Phlebotomine specific cytochrome-b PCR [13].

#### Data analysis

Data analysis was performed using statistical package for social sciences (SPSS) software version 13.5. Sero-prevalence and PCR positivity were calculated in frequency and proportion with rK39 RDT, DAT and PCR positive in all individuals, excluding the past and current VL cases.

## Results

### Characteristics of study clusters

A total of 1,978 residents were recorded during house-to-house surveys from 19 clusters in seven selected VL endemic doubtful districts. A total of 1,682 individuals (85.0%) agreed to participate in the survey; 1,354 individuals (68.5% of the total population) also provided a blood sample. The majority of participants providing a blood sample was female (58.7% (n = 795) females versus 41.3% (n = 559) males), likely linked to the fact that most of the males were out of home for daily labor and economic migration during the survey. Altitude of the clusters ranged from 136 m above sea level in one of the lowland districts to 1,764 m asl in a mountainous district. The characteristics of these study clusters are shown in Table 1.

**Table 1:** Characteristics of the study clusters in selected seven endemic doubtful districts in 2019.

Districts	Municipal Ward No.	Elevation (m asl)	Population size (HHs survey)	Population enrolled in HHs survey	Blood samples collected	Blood sample coverage
Bajura^γ^		-	319	285	230	72.1% (230/319)
	Budhinand—1	378–712	49	36	23	46.9% (23/49)
	Jagganath—2	536–1392	129	112	94	72.9% (94/129)
	Jagganath—5	717–1381	141	137	113	80.1% (113/141)
Banke^α^		-	315	261	206	65.4% (206/315)
	Mahendrapur—2	157–168	161	127	108	67.1% (108/161)
	Puraina—21	136–152	154	134	98	63.6% (98/154)
Bardiya^α^		-	274	242	199	72.6% (199/274)
	Baida—10	140–156	118	111	100	84.7% (100/118)
	Santipur—5	139–153	156	131	99	63.5% (99/156)
Dailekh^β^		-	201	166	129	64.2% (129/201)
	Bhairabi—5	1,181–1,269	62	58	33	53.2% (33/62)
	Dullu—11	960–1,299	95	78	69	72.6% (69/95)
	Guranse—4	1,182–1,270	44	30	27	61.4% (27/44)
Dang^α^		-	359	307	229	63.8% (229/359)
	Dhanauri—3	512–552	118	103	78	66.1% (78/118)
	Lamahi—5	245–248	112	91	69	61.6% (69/112)
	Tulashipur—5	620–650	129	113	82	63.6% (82/129)
Kalikot^*γ*^		-	268	229	187	69.8% (187/268)
	Baitee—8	1,701–1,740	58	46	39	67.2% (39/58)
	Fukot—5	1,735–1,764	103	91	79	76.7% (79/103)
	Raskot—8	1,658–1,693	107	92	69	64.5% (69/107)
Pyuthan^β^		-	242	192	174	71.9% (174/242)
	Airawati—4	632–699	68	52	46	67.6% (46/68)
	Airawati—6	1,030–1,047	126	102	99	78.6% (99/126)
	Swargadwari—9	701–715	48	38	29	60.4% (29/48)
TOTAL			1,978	1,682	1,354	68.5% (1354/1978)

Note: Geographical regions; α - Terai, β - Hilly, γ - Mountainous

### Epidemiological findings

A total of 13 individuals reported having fever ≥ 2 weeks at the time of the survey visit, of whom six had palpable spleen and tested positive to rK39 RDT (five from Bajura and one from Pyuthan districts), thereby meeting the case definition of a current VL case. All six new VL cases were children between five and 14 years of age, three boys and three girls, without a history of travel outside the district. All of them except one (5/6) had a positive DAT result and four out of six were confirmed by PCR. No individuals with skin lesions suspicious for PKDL or cutaneous leishmaniasis (CL) were found during the household survey. A total of 28 past VL cases were ascertained retrospectively: 18 male and 10 female with median age of 28 years (Q1 = 17 years, Q3 = 42 years), ranging from 3 to 66 years at the time of diagnosis. Of the 28 VL cases, 26 (92.9%) had occurred within the three years prior to the study, with the first case reported in 2015. All these past VL cases were rK39 RDT and DAT positive at the time of the study; however, none were PCR positive. Only six of them reported a history of travel to known VL endemic districts in the two years prior to the diagnosis. An additional six individuals were identified as VL deaths; three of them had died during treatment for VL and all were under the age of 20 years.

### Serological and molecular evidence for asymptomatic *Leishmania donovani* infection

**Fig 2:**
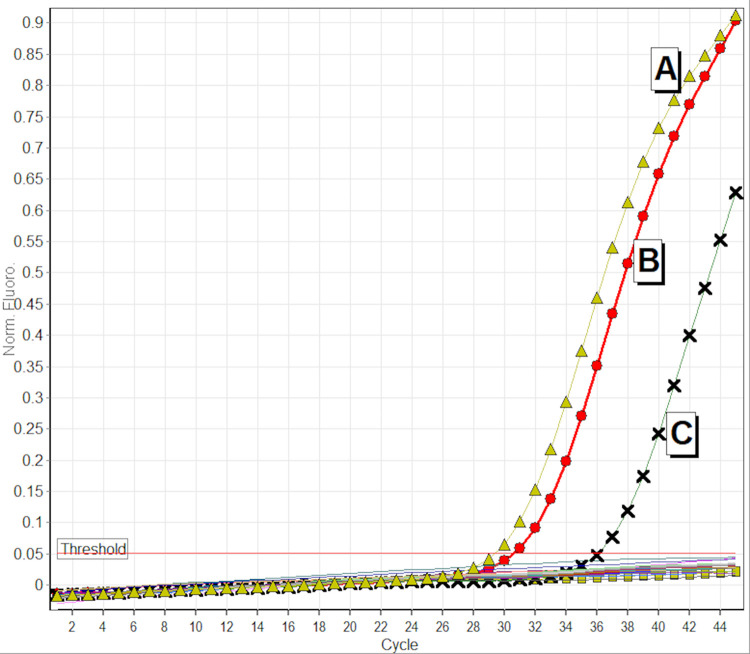
Amplification curve of *Leishmania donovani* complex specific real time PCR. **A:** Sample with *Leishmania donovani* positive PCR result (-Δ-), **B:** Positive Control with 1pg *Leishmania donovani* DNA (-ο-), **C:** Positive Control with 0.1 pg *Leishmania donovani* DNA (-X-), Negative control (-□-).

Serological and molecular results were obtained for 1,320 (97.5%) of 1,354 individuals without past history of VL (Table 2 and S1 Table). Thirty-four blood samples were not tested due to contamination or mix up of the samples during the transportation from field to the laboratory. Overall, 4.1% (54/1,320) of individuals without VL history were positive (asymptomatic infections) on at least one test (rK39 RDT, DAT or PCR), ranging between 2.2% (5/226) in Dang to 6.0% (13/218) in Bajura. Asymptomatic infection was 3% (6/203) in Banke, 4.0% (5/126) in Dailekh, 4.1% (8/197) in Bardiya, 4.2% (7/168) in Pyuthan and 5.5% (10/182) in Kalikot districts. Median age of these asymptomatically infected individuals was 16 years (Q1 = 11, Q3 = 23); 57% (31/54) were male (S1 Data). None had a travel history outside their district of residence in the two years prior to the survey. Seventeen percent (9/54) of asymptomatically infected individuals reported a history of VL among their household members, compared to 0.5% (6/1,266) among individuals without any positive marker of infection. SSU-rDNA PCR for *L*. *donovani complex* was confirmed in two asymptomatic individuals (Fig 2).

**Table 2:** Prevalence of rK39 RDT, DAT and PCR positive cases including sand fly status in seven endemic doubtful districts in western Nepal (n = 1320).

Districts	No. of VL deaths	No. of VL cases ascertained (past + current)	No. VL cases-no travel (past + current)	Excluding past and current VL cases			*P*. *argentipes* present	*P*. *argentipes* infected with *L*. *donovani*
				rk39 RDT positive % (n/N)	DAT positive % (n/N)	PCR positive % (n/N)		
Bajura	3	7+ 5* = 13	7 + 5* = 12	1.8% (4/218)	4.1% (9/218)	3.2% (7/218)	No	No
Banke	0	3 + 0* = 3	1 + 0* = 1	1.5% (3/203)	2.9% (6/203)	1.5% (3/203)	Yes	No
Bardiya	1	2 + 0* = 2	2 + 0* = 2	1.5% (3/197)	4.1% (8/197)	2.0% (4/197)	Yes	No
Dailekh	0	3 + 0* = 3	0 + 0* = 0	2.4% (3/126)	3.2% (4/126)	2.4% (3/126)	Yes	No
Dang	0	3 + 0* = 3	2 + 0* = 2	1.0% (2/226)	2.2% (5/226)	1.3% (3/226)	Yes	No
Kalikot	2	5 + 0* = 5	5 + 0* = 5	2.7% (5/182)	4.4% (8/182)	3.8% (7/182)	Yes	No
Pyuthan	0	5 + 1* = 6	5 + 1* = 6	2.4% (4/168)	4.2% (7/168)	3.6% (6/168)	Yes	No
TOTAL	6	28 + 6* = 35	22 + 6* = 28	1.8% (24/1,320)	3.6% (47/1,320)	2.5% (33/1,320)		

*New VL case

### Entomological findings

The total numbers of 711 sand flies were captured from all 19 surveyed clusters. Sand flies were classified into *P*. *argentipes*, *P*. *papatasi*, *P*. *major* sensu lato, *P*. *(Adlerius)* spp. and *Sergentomyia* spp. The incriminated vector species for *L*. *donovani* in Nepal, *P*. *argentipes* was found in all districts except Bajura and captured at an elevation up to 1,764 m asl. Other Phlebotomine sand flies; *P*. *major* s.l. and *P*. (*Adlerius*) spp. were documented from Bajura, Dailekh, Pyuthan and Kalikot districts at altitudes ranging between 378–1764 m asl. Out of 134 pools of sand flies assessed, only one was positive for *L*. *donovani* and this pool was belonged to *Phlebotomus (Adlerius)* spp. None of the pools of *P*. *argentipes* was found infected with the parasite. Tracing the origin of the positive pool of sand flies, these were captured from a single house of the study cluster in Bajura district at an altitude of 1,279 m asl. This household was neighboring to a household with a past and present VL cases. Entomological findings per sand fly species and sex are summarized in S2 Table.

## Discussion

We assessed the evidence of local transmission of *L*. *donovani* in seven districts of doubtful VL endemicity in the western part of Nepal. In all districts except for one, the majority of VL cases identified did not have a history of travel to known VL endemic areas in the two years prior to the start of their VL symptoms. In addition, none of the identified asymptomatic infections during the survey had a such travel history. Seventeen percent of these asymptomatically infected persons reported at least one VL case in the past among their household members, compared to 0.5% of non-infected participants. Also, the vector sand fly species, *P*. *argentipes* was present in all districts except one and up to an altitude as high as 1,764 m asl. Although none of the *P*. *argentipes* pools examined were found positive for *L*. *donovani*, the combined evidence points towards local transmission of the parasite in all selected districts.

This, to our knowledge, is the first extensive transmission assessment survey conducted in western Nepal to objectify the observed geographical spread of reported VL cases in recent years. There are some limitations to our study. Importantly, the use of serological and molecular tests as markers for asymptomatic infection is suboptimal. None of the markers used (rK39 RDT, DAT and PCR) have been validated as a standalone marker of infection. Likely, each marker represents a different subgroup or stage of infection. By combining several tests, we have attempted to increase the sensitivity for the detection of infection. Specificity of the serological tests used appears to be excellent. Studies in India performing the rK39 RDT and DAT on apparently healthy (non-VL diseased) individuals from non-endemic areas, did not report any positive results, suggesting a specificity close to 100% for both tests [14, 15].

The tests to detect asymptomatic infection in the human population were selected to identify infection with *L*. *donovani*, as this is assumed to be the only causative species for VL in Nepal. However, other parasite species that may occasionally cause VL have also been reported in the region, such as *L*. *tropica* in neighboring India [16]. More recently, *L*. *major* was reported as one of the causative species for cutaneous leishmaniasis cases in Nepal [17]. This species has shown the ability to visceralize in an Iranian settings [18]. If the circulating parasite species causing VL in these previously non-endemic districts in Nepal is different from *L*. *donovani*, the seroprevalence results from the tests used to identify asymptomatic infection might be an important underestimation of the true transmission levels in the areas assessed.

We found that 3.6% of individuals without a VL history were positive with DAT. This is similar to the DAT seroprevalence of 4.4% reported from highly endemic clusters in the Eastern *Terai* in 2010 [19] and lower than the 9.3% reported from Eastern hills in 2015 [4]. The relatively low seroprevalence found in this study reflects the lower VL incidence rate reported in the western part of Nepal compared to the incidence rates reported previously in the eastern hilly or *Terai* region. This might be a reflection of recent spread of the infection to the study districts. The positivity rates among individuals without a VL history found in our study using the rK39 RDT and PCR (1.7% and 2.5% respectively) are comparable with those found in a recent study conducted in the program district (Palpa) of western Nepal [20].

We found the sand fly vector, *P*. *argentipes* at altitudes up to 1,764 m asl. This is considerably higher than the 600 m limit that was historically assumed to be the maximum altitude for vector survival [20–22]. Nonetheless, our findings are supported by other studies in Nepal as well [23]. As *P*. *argentipes* sand flies were present in all districts except one, our findings support its position as the potential vector of *L*. *donovani* in these regions. The fact that none of the *P*. *argentipes* pools were found infected with *L*. *donovani* does not refute its likely role as a vector, as even in highly endemic foci in Nepal and neighboring India, the infection rates in sand flies are very low [24–26].

Also the *Phlebotomine* species *P*. *major* and *P*. *(Adlerius)* spp. were collected in the hilly and mountainous regions of Bajura, Dailekh, Kalikot and Pyuthan districts from altitudes ranging from 378 to 1,764 m asl. This is the very first report on these species in recent years from the western part of Nepal. Nonetheless, similar species were also observed to be present at high altitudes (947–2,130 m) in the bordering state of India [27, 28]. Interestingly, we demonstrated the presence of *L*. *donovani* DNA in a pool of *P*. *(Adlerius)* spp. One of the confirmed species from the same subgenus, *P*. *(Adlerius) longiductus* was found naturally infected with *L*. *donovani* and suspected to be the vector involved in cutaneous leishmaniasis transmission in Himachal Pradesh (India) in the north-western Himalaya. In this area VL and CL co-exist [28], as is the case in the western part of Nepal in recent years. Although the presence of *L*. *donovani* DNA in *Phlebotomus (Adlerius)* spp. does not prove their role as a vector, this finding does warrant further exploration to assess this possibility.

As the entomological survey was cross-sectional, we could not collect information on the actual diversity and seasonality of the sand fly species in these diverse geographical areas. Such information is possible only if we conduct longitudinal entomological collections. It is therefore possible that in Bajura, the only district where *P*. *argentipes* was not found during our survey, the vector is indeed present at another time point during the year. Without prior knowledge about the seasonality of vectors in mountainous districts such as Bajura, however, the perfect timing of vector collections remains a challenge.

In recent years, cutaneous leishmaniasis (CL) has emerged as a public concern in western Nepal, despite the country originally not being known as endemic for this disease. A recent study reported both *L*. *donovani* and *L*. *major* as causative species for CL in Nepal [17]. If *L*. *donovani* is indeed implicated in CL in Nepal, CL patients could form a threat for VL elimination by constituting a reservoir of which the extent is largely unknown at present. In addition, *L*. *major* has illustrated its ability to visceralize and cause VL as well [18]. This could have implications for species-specific diagnostic and treatment needs for VL patients. Therefore, rigorous surveillance as well as further exploration of circulating *Leishmania* spp. and potentially implicated vectors for both VL and CL will be of paramount importance to adjust the ongoing VL elimination efforts to the changing reality.

In conclusion, the study suggests that there is local transmission of *L*. *donovani* in the seven VL endemic doubtful districts in western Nepal. Policy makers should revise the current endemicity status for VL reconsidering whether the strict distinction between endemic, endemic doubtful and non-endemic districts, as currently defined in the National Guidelines [2] is still appropriate. At present, the demonstration of a full transmission cycle is still a crucial criterion for declaring a district as endemic. More recently, however, the Regional Technical Advisory Group Meeting for VL elimination program 2022 has suggested that presence of one locally acquired VL case in the last 10 years in combination with presence of a competent vector should suffice to declare endemicity [29]. With the vector present at altitudes up to 1,700 m asl as illustrated in this study, the likelihood is high that many other districts currently labelled endemic doubtful or non-endemic also have ongoing local transmission. Rather than repeating resource-intensive transmission assessments in each of the remaining districts in Nepal, it might be more rational to ensure that the basics for disease control interventions are present in all districts, whether officially designated endemic for VL or not. Making diagnostic tests widely available in all endemic doubtful districts and ensuring that all diagnosed cases are included in the surveillance and reporting system will be a crucial first step towards a better understanding of the true extent of VL endemicity in Nepal, without which disease elimination is difficult to achieve and sustain.

## Supporting information

S1 Fig VL reported in Nepal between 2007–2021. (TIF)

S1 Table Prevalence of rK39 RDTs DAT and PCR positive cases. (DOCX)

S2 Table Entomological findings per sandfly species and sex. (DOCX)

S1 Data Visceral leishmaniasis in the hills of western Nepal- a transmission. (XLSX)

## References

[1] World Health Organization. Regional Strategic Framework for accelerating and sustaining elimination of kala-azar in the South-East Asia Region: 2022–2026. World Health Organization, Regional Office for South-East Asia, New Delhi: World Health Organization; 2022.

[2] Epidemiology and Disease Control Division. National Guideline on Kala-azar Elimination Program (Updated) 2019. Department of Health Services, Ministry of Health and Population, Government of Nepal, Teku, Kathmandu. 2019.

[3] Pandey BD, Pun SB, Kaneko O, Pandey K, Hirayama K. Case report: Expansion of visceral leishmaniasis to the western hilly part of Nepal. Am J Trop Med Hyg. 2011;84(1):107–8. 10.4269/ajtmh.2011.10–0291 doi: 10.4269/ajtmh.2011.10-0291 21212211 PMC3005498

[4] Ostyn B, Uranw S, Bhattarai NR, Das ML, Rai K, Tersago K, et al. Transmission of Leishmania donovani in the Hills of Eastern Nepal, an Outbreak Investigation in Okhaldhunga and Bhojpur Districts. PLoS Negl Trop Dis. 2015;9(8):e0003966. doi: 10.1371/journal.pntd.0003966 26252494 PMC4529159

[5] Epidemiology and Disease Control Division. Report of the Experts’ Consultation and Program Review. Kala-azar Elimination Program. 2017.

[6] Picado A, Das ML, Kumar V, Dinesh DS, Rijal S, Singh SP, et al. Phlebotomus argentipes seasonal patterns in India and Nepal. J Med Entomol. 2010;47(2):283–6. doi: 10.1603/me09175 20380311

[7] Kalra NL, Bang YH. Manuals on Entomology in Visceral Leishmaniasis: World Health Organization, SEARO, New Delhi; 1988.

[8] Lewis DJ. A taxonomic review of the genus *Phlebotomus* (Diptera: Psychodidae). Bulletin of British Museum of Entomology (Natural History). 1982;45:121–209.

[9] Jacquet D, Boelaert M, Seaman J, Rijal S, Sundar S, Menten J, et al. Comparative evaluation of freeze-dried and liquid antigens in the direct agglutination test for serodiagnosis of visceral leishmaniasis (ITMA-DAT/VL). Trop Med Int Health. 2006;11(12):1777–84. 10.1111/j.1365-3156.2006.01743.x doi: 10.1111/j.1365-3156.2006.01743.x 17176341

[10] Khanal B, Picado A, Bhattarai NR, Van Der Auwera G, Das ML, Ostyn B, et al. Spatial analysis of Leishmania donovani exposure in humans and domestic animals in a recent kala azar focus in Nepal. Parasitology. 2010;137(11):1597–603. 10.1017/S0031182010000521 doi: 10.1017/S0031182010000521 20459877

[11] Deborggraeve S, Boelaert M, Rijal S, De Doncker S, Dujardin JC, Herdewijn P, et al. Diagnostic accuracy of a new Leishmania PCR for clinical visceral leishmaniasis in Nepal and its role in diagnosis of disease. Trop Med Int Health. 2008;13(11):1378–83. 10.1111/j.1365-3156.2008.02154.x doi: 10.1111/j.1365-3156.2008.02154.x 18803611

[12] Merdekios B, Pareyn M, Tadesse D, Eligo N, Kassa M, Jacobs BKM, et al. Evaluation of conventional and four real-time PCR methods for the detection of Leishmania on field-collected samples in Ethiopia. PLoS Negl Trop Dis. 2021;15(1):e0008903. 10.1371/journal.pntd.0008903 doi: 10.1371/journal.pntd.0008903 33434190 PMC7802924

[13] Esseghir S. Speciation of Phlebotomus sandflies of the subgenus Larroussius coincided with the late Miocene-Pliocene aridification of the Mediterranean subregion. Biol J Linn Soc. 2000;70:189–219. 10.1006/bijl.1999.0393

[14] Sundar S, Singh RK, Maurya R, Kumar B, Chhabra A, Singh V, et al. Serological diagnosis of Indian visceral leishmaniasis: direct agglutination test versus rK39 strip test. Trans R Soc Trop Med Hyg. 2006;100(6):533–7. 10.1016/j.trstmh.2005.08.018 doi: 10.1016/j.trstmh.2005.08.018 16325874

[15] Cloots K, Singh OP, Singh AK, Kushwaha AK, Malaviya P, Kansal S, et al. Diagnosis of Visceral Leishmaniasis in an Elimination Setting: A Validation Study of the Diagnostic Algorithm in India. Diagnostics (Basel). 2022;12(3). 10.3390/diagnostics12030670 10.3390/diagnostics12030670 PMC8947297 35328223

[16] Khanra S, Datta S, Mondal D, Saha P, Bandopadhyay SK, Roy S, et al. RFLPs of ITS, ITS1 and hsp70 amplicons and sequencing of ITS1 of recent clinical isolates of Kala-azar from India and Bangladesh confirms the association of L. tropica with the disease. Acta Trop. 2012;124(3):229–34. 10.1016/j.actatropica.2012.08.017 doi: 10.1016/j.actatropica.2012.08.017 22960646

[17] Rai T, Shrestha S, Prajapati S, Bastola A, Parajuli N, Ghimire PG, et al. Leishmania donovani persistence and circulation causing cutaneous leishmaniasis in unusual-foci of Nepal. Sci Rep. 2023;13(1):12329. 10.1038/s41598-023-37458-6 doi: 10.1038/s41598-023-37458-6 37516780 PMC10387047

[18] Karamian M, Motazedian MH, Mehrabani D, Gholami K. Leishmania major infection in a patient with visceral leishmaniasis: treatment with Amphotericin B. Parasitol Res. 2007;101(5):1431–4. 10.1007/s00436-007-0649-x doi: 10.1007/s00436-007-0649-x 17659388

[19] Rijal S, Uranw S, Chappuis F, Picado A, Khanal B, Paudel IS, et al. Epidemiology of Leishmania donovani infection in high-transmission foci in Nepal. Trop Med Int Health. 2010;15 Suppl 2:21–8. 10.1111/j.1365-3156.2010.02518.x 20487421

[20] Basnyat S, Banjara MR, Ghimire P, Matlashewski G, Singh A. Seropositivity of Visceral leishmaniasis on people of VL endemic three districts of Nepal. Parasitol Int. 2021;80:102236. 10.1016/j.parint.2020.102236 doi: 10.1016/j.parint.2020.102236 33147500

[21] Park K. Leishmaniasis in K Park-Park’s (ed.) The Textbook of Preventive and Social Medicine. 6 ed. Jabalpur, India2015 2015.

[22] Bhunia GS, Kesari S, Jeyaram A, Kumar V, Das P. Influence of topography on the endemicity of Kala-azar: a study based on remote sensing and geographical information system. Geospat Health. 2010;4(2):155–65. 10.4081/gh.2010.197 doi: 10.4081/gh.2010.197 20503185

[23] Lysenko AJ. Distribution of leishmaniasis in the Old World. Bull World Health Organ. 1971;44(4):515–20. 5316978 PMC2427824

[24] Roy L, Cloots K, Uranw S, Rai K, Bhattarai NR, Smekens T, et al. The ongoing risk of Leishmania donovani transmission in eastern Nepal: an entomological investigation during the elimination era. Parasites & Vectors. 2023;16(1):404. 10.1186/s13071-023-05986-9 doi: 10.1186/s13071-023-05986-9 37932813 PMC10629032

[25] Deb R, Singh RP, Mishra PK, Hitchins L, Reid E, Barwa AM, et al. Impact of IRS: Four-years of entomological surveillance of the Indian Visceral Leishmaniases elimination programme. PLoS Negl Trop Dis. 2021;15(8):e0009101. 10.1371/journal.pntd.0009101 doi: 10.1371/journal.pntd.0009101 34370731 PMC8376195

[26] Bhattarai NR, Das ML, Rijal S, van der Auwera G, Picado A, Khanal B, et al. Natural infection of Phlebotomus argentipes with Leishmania and other trypanosomatids in a visceral leishmaniasis endemic region of Nepal. Trans R Soc Trop Med Hyg. 2009;103(11):1087–92. doi: 10.1016/j.trstmh.2009.03.008 19345387

[27] Sharma NL, Mahajan VK, Ranjan N, Verma GK, Negi AK, Mehta KI. The sandflies of the Satluj river valley, Himachal Pradesh (India): some possible vectors of the parasite causing human cutaneous and visceral leishmaniases in this endemic focus. J Vector Borne Dis. 2009;46(2):136–40. 19502693

[28] Lata S, Kumar G, Ojha VP, Dhiman RC. Detection of Leishmania donovani in Wild-Caught Phlebotomine Sand Flies in Endemic Focus of Leishmaniasis in Himachal Pradesh, India. J Med Entomol. 2022;59(2):719–24. 10.1093/jme/tjab202 doi: 10.1093/jme/tjab202 34865089 PMC8924965

[29] World Health Organization. Regional Office for South-East Asia. Report of the meeting of programme managers and the Regional Technical Advisory Group for kala-azar elimination programme, virtual meeting, 18–20 April 2022. WHO, Regional Office for South-East Asia; 2022.

