# Supplementary figures and images for: Visceral leishmaniasis in the hills of western Nepal: A transmission assessment
Source: PLoS One. 2024 Apr 17;19(4):e0289578. doi: 10.1371/journal.pone.0289578 (PMC11023194; doi:10.1371/journal.pone.0289578)

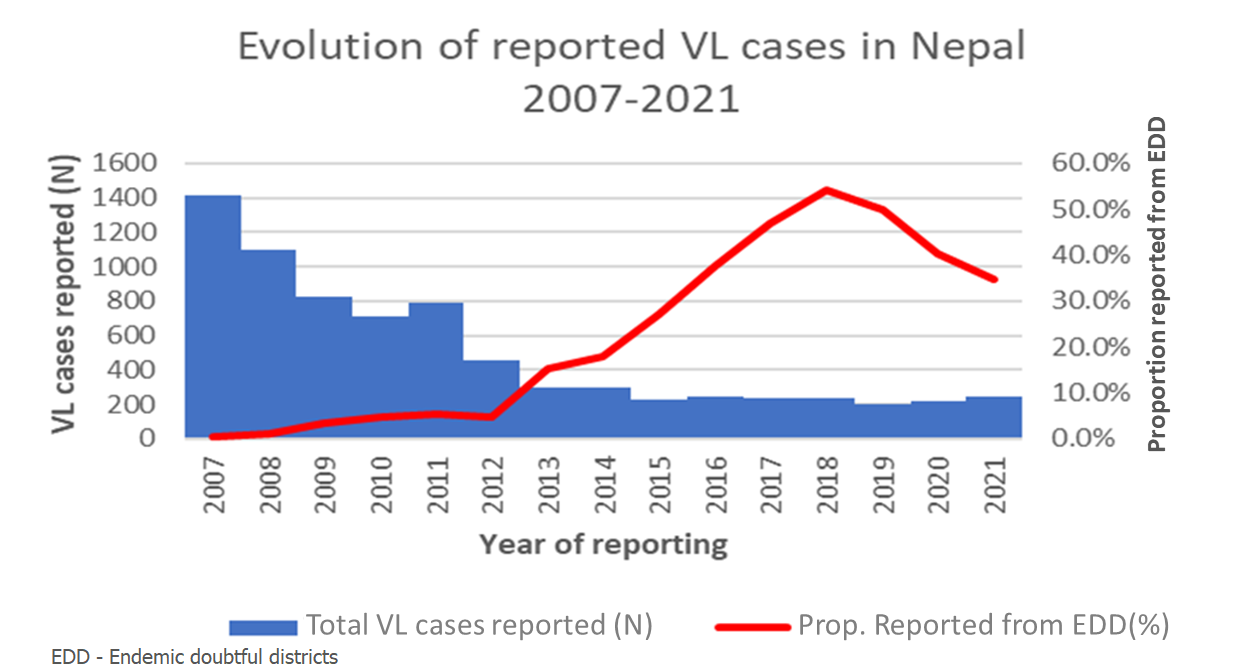

Supplement: S1 Fig — (TIF) [file pone.0289578.s001.tif]
