# Supplementary material for: Visceral leishmaniasis in the hills of western Nepal: A transmission assessment
Source: PLoS One. 2024 Apr 17;19(4):e0289578. doi: 10.1371/journal.pone.0289578 (PMC11023194; doi:10.1371/journal.pone.0289578)
Supplement: S1 Table — (DOCX) [file pone.0289578.s002.docx]

**S1 Table.** Demography, Serosurvey and results of VL diagnostic tests in 19 surveyed clusters in seven endemic doubtful districts in western Nepal in 2019

| Districts | Municipality-Ward No. | Altitude  (m asl) | Coverage | Blood Samples collected | Population present | Population size | No. of VL deaths | VL cases ascertained (old + new) | | No.of VL case-no travel history (past + current) | **Excluding past and current VL cases** | | | | | *P*. argentipes present |  |  |
| --- | --- | --- | --- | --- | --- | --- | --- | --- | --- | --- | --- | --- | --- | --- | --- | --- | --- | --- |
|  |  |  |  |  |  |  |  |  |  |  | Rk39 RDT Positive  % (n/N) | DAT Positive  % (n/N) | | PCR Positive  % (n/N) | |  |  |  |
| **Bajura** |  |  | **72.1% (230/319)** | **230** | **285** | **319** | **3** | **7 + 5* = 12** | | **7 + 5* = 12** | **1.4% (3/218)** | **4.1% (9/218)** | | **3.2% (7/218)** | |  | |  |
|  | Budhinand - 1 | 378 - 712 | 46.9% (23/49) | 23 | 36 | 49 | 0 | 1 + 1* = 2 | | 1 + 1* = 2 | 0% (0/36) | 3% (1/36) | | 0% (0/36) | | No | |  |
|  | Jagganath - 2 | 536 - 1392 | 72.9% (94/129) | 94 | 112 | 129 | 2 | 3 + 4* = 7 | | 3 + 4* = 7 | 0% (0/83) | 4% (3/83) | | 4% (3/83) | | No | |  |
|  | Jagganath - 5 | 717 - 1381 | 80.1% (113/141) | 113 | 137 | 141 | 1 |  | 3 + 0* = 3 | 3 + 0* = 3 | 3% (3/99) | 5% (5/99) | | 4% (4/99) | | No |  |  |
| **Banke** |  |  | **65.4% (206/315)** | **206** | **261** | **315** | **0** | **3 + 0* = 3** | | 1 + 0* = 1 | **1.5% (3/203)** | **3% (6/203)** | | **1.5% (3/203)** | |  | | |
|  | Mahendrapur - 2 | 157 - 168 | 67.1% (108/161) | 108 | 127 | 161 | 0 | 1 + 0* = 1 | | 1 + 0* = 1 | 1% (1/105) | 2% (2/105) | | 1% (1/105) | | Yes | | |
|  | Puraina - 21 | 136 - 152 | 63.6% (98/154) | 98 | 134 | 154 | 0 | 2 + 0* = 2 | | 0 + 0* = 0 | 2% (2/98) | 4% (4/98) | | 2% (2/98) | | Yes | | |
| **Bardiya** |  |  | **73% (199/274)** | **199** | **242** | **274** | **1** | **2 + 0* = 2** | | **2 + 0* = 2** | **1.5% (3/197)** | **4.1% (8/197)** | | **2.0% (4/197)** | |  | | |
|  | Baida - 10 | 140 - 156 | 86% (101/118) | 100 | 111 | 118 | 1 | 1 + 0* = 1 | | 1 + 0* = 1 | 1% (1/98) | 3% (3/98) | | 2% (2/100) | | Yes | | |
|  | Santipur - 5 | 139 - 153 | 63% (98/156) | 99 | 131 | 156 | 0 | 1 + 0* = 1 | | 1 + 0* = 1 | 2% (2/99) | 5% (5/99) | | 2% (2/99) | | Yes | | |
| **Dailekh** |  |  | **64.2% (129/201)** | **129** | **166** | **201** | **0** | **3 + 0* = 3** | | **0 + 0* = 0** | **2.4% (3/126)** | **3.2% (4/126)** | | **2.4% (3/126)** | |  | | |
|  | Bhairabi - 5 | 1181 - 1269 | 53.2% (33/62) | 33 | 58 | 62 | 0 | 1 + 0* = 1 | | 0 + 0* = 0 | 3% (1/32) | 9% (3/32) | | 6% (2/32) | | Yes | | |
|  | Dullu - 11 | 960 - 1299 | 72.6% (69/95) | 69 | 78 | 95 | 0 | 1 + 0* = 1 | | 0 + 0* = 0 | 1% (1/68) | 0% (0/68) | | 0% (0/68) | | Yes | | |
|  | Guranse - 4 | 1182 - 1270 | 61.4% (27/44) | 27 | 30 | 44 | 0 | 1 + 0* = 1 | | 0 + 0* = 0 | 4% (1/26) | 4% (1/26) | | 4% (1/26) | | Yes | | |
| **Dang** |  |  | 63.8% (229/359) | **229** | **307** | **359** | **0** | **3 + 0* = 3** | | **2 + 0 = 2** | **1.0% (2/226)** | **2.2% (5/226)** | | **1.3% (3/226)** | |  | | |
|  | Dhanauri - 3 | 512 - 552 | 66.1% (78/118) | 78 | 103 | 118 | 0 | 1 + 0* = 1 | | 0 + 0* = 0 | 1% (1/77) | 3% (2/77) | | 3% (2/77) | | Yes | | |
|  | Lamahi - 5 | 245 - 248 | 61.6% (69/112) | 69 | 91 | 112 | 0 | 1 + 0* = 1 | | 1 + 0* = 1 | 1% (1/69) | 3% (2/69) | | 2% (1/69) | | Yes | | |
|  | Tulashipur - 5 | 620 - 650 | 63.6% (82/129) | 82 | 113 | 129 | 0 | 1 + 0* = 1 | | 1 + 0* = 1 | 0% (0/80) | 1% (1/80) | | 0% (0/80) | | Yes | | |
| **Kalikot** |  |  | **69.8% (187/268)** | **187** | **229** | **268** | **2** | **5 + 0* = 5** | | **5 + 0* = 5** | **2.7% (5/182)** | **4.4% (8/182)** | | **3.8% (7/182)** | |  | | |
|  | Baitee - 8 | 1701 - 1740 | 67.2% (39/58) | 39 | 46 | 58 | 1 | 1 + 0* = 1 | | 1 + 0* = 1 | 3% (1/38) | 3% (1/38) | | 3% (1/38) | | Yes | | |
|  | Fukot - 5 | 1735 - 1764 | 76.7% (79/103) | 79 | 91 | 103 | 0 | 1 + 0* = 1 | | 1 + 0* = 1 | 3% (2/77) | 4% (3/77) | | 4% (3/77) | | Yes | | |
|  | Raskot - 8 | 1658 - 1693 | 64.5% (69/107) | 69 | 92 | 107 | 1 | 3 + 0* = 3 | | 3 + 0* = 3 | 3% (2/67) | 6% (4/67) | | 4% (3/67) | | Yes | | |
| **Pyuthan** |  |  | **71.9% (174/242)** | **174** | **192** | **242** | **0** | **5 + 1* = 6** | | **5 + 1* = 6** | **2.4% (4/168)** | **4.2% (7/168)** | | **3.6% (6/168)** | |  | | |
|  | Airawati - 4 | 632 – 699 | 67.6% (46/68) | 46 | 52 | 68 | 0 | 2 + 1* = 3 | | 2 + 1* = 3 | 0% (0/44) | 5% (2/44) | | 2% (1/46) | | Yes | | |
|  | Airawati - 6 | 1030 - 1047 | 78.6% (99/126) | 99 | 102 | 126 | 0 | 2 + 0* = 2 | | 2 + 0* = 2 | 3% (3/97) | 4% (4/97) | | 4% (4/99) | | Yes | | |
|  | Swargadwari - 9 | 701 - 715 | 60.4% (29/48) | 29 | 38 | 48 | 0 | 1 + 0* = 1 | | 1 + 0* = 1 | 4% (1/27) | 4% (1/27) | | 3% (1/29) | | Yes | | |
|  | TOTAL |  | 68.5% (1,354/1,978) | 1,354 | 1,682 | 1,978 | 6 | 28 + 6* = 34 | | 22 +6* = 28 | 1.7% (23/1,320) | | 3.6% (47/1,320) | 2.5% (33/1,320) |  | | | |
