# Supplementary material for: Visceral leishmaniasis in the hills of western Nepal: A transmission assessment
Source: PLoS One. 2024 Apr 17;19(4):e0289578. doi: 10.1371/journal.pone.0289578 (PMC11023194; doi:10.1371/journal.pone.0289578)
Supplement: S2 Table — (DOCX) [file pone.0289578.s003.docx]

**S2 Table.** Entomological findings per sand fly species and sex by survey districts and clusters in 2019

| **Districts/**  **Survey clusters** | **Villages** | **Phlebotomine sand flies** | | | | | | | | | | |
| --- | --- | --- | --- | --- | --- | --- | --- | --- | --- | --- | --- | --- |
|  |  | ***P. argentipes*** | | ***P. papatasi*** | | ***P. major*** | | ***P.* (*Adlerius*) spp.** | | ***P. sergenti*** | | **Total** |
|  |  | Male | Female | Male | Female | Male | Female | Male | Female | Male | Female |  |
| **Bajura district** | | **0** | **0** | **0** | **0** | **55** | **32** | **60** | **93** | **0** | **0** | **240** |
| Budinanda - 1 | Pipaldali | 0 | 0 | 0 | 0 | 5 | 2 | 0 | 1 | 0 | 0 | 8 |
| Jaggnath - 2 | Bamanjiula | 0 | 0 | 0 | 0 | 4 | 7 | 21 | 46 | 0 | 0 | 78 |
| Jaggnath - 5 | Baksena | 0 | 0 | 0 | 0 | 46 | 23 | 39 | 46 | 0 | 0 | 154 |
| **Banke district** | | **44** | **52** | **3** | **7** | **0** | **0** | **0** | **0** | **0** | **0** | **106** |
| Mahendrapur - 2 | Mahendranagar | 33 | 29 | 0 | 5 | 0 | 0 | 0 | 0 | 0 | 0 | 67 |
| Puraina - 21 | Puraina | 11 | 23 | 3 | 2 | 0 | 0 | 0 | 0 | 0 | 0 | 39 |
| **Bardiya district** | | 21 | 54 | 1 | 2 | 0 | 0 | 0 | 0 | 0 | 0 | **78** |
| Baida - 10 | Dhadhwar | 4 | 25 | 0 | 0 | 0 | 0 | 0 | 0 | 0 | 0 | 29 |
| Santipur - 5 | Santipur | 17 | 29 | 1 | 2 | 0 | 0 | 0 | 0 | 0 | 0 | 49 |
| **Dailekh district** | | **7** | **13** | **0** | **0** | **41** | **3** | **7** | **0** | **0** | **0** | **71** |
| Bhairabi - 5 | Bhairabi | 0 | 0 | 0 | 0 | 21 | 1 | 5 | 0 | 0 | 0 | 27 |
| Dullu - 11 | Simli | 7 | 13 | 0 | 0 | 20 | 2 | 1 | 0 | 0 | 0 | 43 |
| Guranse - 4 | Salghari tole | 0 | 0 | 0 | 0 | 0 | 0 | 1 | 0 | 0 | 0 | 1 |
| **Dang district** | | **7** | **4** | **2** | **0** | **0** | **0** | **0** | **0** | **4** | **2** | **19** |
| Dhanauri - 3 | Dhanaura | 7 | 4 | 0 | 0 | 0 | 0 | 0 | 0 | 3 | 2 | 16 |
| Lamahi - 5 | Lamahi | 0 | 0 | 2 | 0 | 0 | 0 | 0 | 0 | 1 | 0 | 3 |
| Tulsipur - 5 | Samjhana tole | 0 | 0 | 0 | 0 | 0 | 0 | 0 | 0 | 0 | 0 | 0 |
| **Kalikot district** | | **0** | **6** | **0** | **0** | **25** | **14** | **9** | **16** | **0** | **0** | **70** |
| Baitee - 8 | Paltibada | 0 | 0 | 0 | 0 | 6 | 3 | 3 | 5 | 0 | 0 | 17 |
| Fukot - 5 | Fuku | 0 | 4 | 0 | 0 | 15 | 4 | 1 | 6 | 0 | 0 | 30 |
| Raskot - 8 | Ranagaun | 0 | 2 | 0 | 0 | 4 | 7 | 5 | 5 | 0 | 0 | 23 |
| **Pyuthan district** | | **25** | **70** | **4** | **0** | **6** | **6** | **6** | **10** | **0** | **0** | **127** |
| Airavati - 4 | Khamchi | 20 | 59 | 3 | 0 | 2 | 1 | 0 | 0 | 0 | 0 | 85 |
| Airavati - 6 | Airikhola | 1 | 6 | 0 | 0 | 2 | 5 | 6 | 9 | 0 | 0 | 29 |
| Swargadwari - 9 | Bhajeni | 4 | 5 | 1 | 0 | 2 | 0 | 0 | 1 | 0 | 0 | 13 |
